# Prediction of protein binding residues for metal ions, nucleic acids, and small molecules

**DOI:** 10.1093/bib/bbaf631.068

**Published:** 2025-12-12

**Authors:** Nguyen Quoc Khanh Le

**Affiliations:** In-Service Master Program in Artificial Intelligence in Medicine, College of Medicine, Taipei Medical University, Taipei 110, Taiwan; AIBioMed Research Group, Taipei Medical University, Taipei 110, Taiwan

## Abstract

**Background:**

Protein–ligand interactions are central to cellular regulation and therapeutic targeting. Experimental identification of binding residues remains costly and time-consuming, motivating the need for efficient computational approaches. We present an interpretable, XGBoost-based framework for residue-level prediction of protein binding sites specific to three ligand classes—metal ions, nucleic acids, and small molecules—emphasizing predictive accuracy and user accessibility.

**Methods:**

A benchmark dataset [1] comprising 1314 annotated protein sequences was processed into peptide windows (±5, ±11, ±20 residues) centered on binding sites, maintaining a 1:3 ratio of positive to negative samples. Sequence-derived descriptors were generated using the iLearn toolkit, including amino acid composition (AAC), grouped AAC (GAAC), CTD composition, and BLOSUM62 substitution scores, yielding >8000 features subsequently reduced by feature-importance heuristics. More than 60 classifiers were evaluated; XGBoost consistently achieved superior performance after hyperparameter optimization.

**Results:**

Independent models trained for each ligand type achieved high predictive power and biological interpretability. For small-molecule binding, the model attained an AUC of 0.99 and F1 of 0.91, with key features involving small residues (G, C, S, A) reflecting steric constraints. The nucleic-acid model reached AUC 0.99 and F1 0.95, highlighting glycine-rich and positively charged motifs typical of RNA/DNA interactions. The metal-ion model (AUC 0.92, F1 0.65) emphasized cysteine and histidine, consistent with metalloprotein binding patterns. Compared with reference CNN models, our framework achieved lower log-loss, greater interpretability, and stable cross-validation.

**Conclusion:**

A standalone graphical user interface (GUI) implemented in Python allows users to input protein sequences and obtain residue-level binding probabilities, exportable as CSV files. This work demonstrates that well-engineered classical machine learning can rival deep learning in protein binding prediction, offering interpretable, high-performance tools to support biomedical and pharmaceutical research.

**References:**

[1] Littmann M, Heinzinger M, Dallago C, Weissenow K, Rost B. Protein embeddings and deep learning predict binding residues for various ligand classes. *Sci Rep*. 2021 Dec 13;11(1):23916.

